# Recent Development of Supramolecular Cancer Theranostics Based on Cyclodextrins: A Review

**DOI:** 10.3390/molecules28083441

**Published:** 2023-04-13

**Authors:** Wenting Hu, Binglin Ye, Guocan Yu, Feihe Huang, Zhengwei Mao, Yuan Ding, Weilin Wang

**Affiliations:** 1 Department of Hepatobiliary and Pancreatic Surgery, The Second Affiliated Hospital, Zhejiang University School of Medicine, Hangzhou 310009, China; huwt2020@zju.edu.cn (W.H.);; 2 Key Laboratory of Precision Diagnosis and Treatment for Hepatobiliary and Pancreatic Tumor of Zhejiang Province, Hangzhou 310009, China; 3 Research Center of Diagnosis and Treatment Technology for Hepatocellular Carcinoma of Zhejiang Province, Hangzhou 310009, China; 4 Clinical Medicine Innovation Center of Precision Diagnosis and Treatment for Hepatobiliary and Pancreatic Disease, Zhejiang University, Hangzhou 310009, China; 5 Clinical Research Center of Hepatobiliary and Pancreatic Diseases of Zhejiang Province, Hangzhou 310009, China; 6 Cancer Center, Zhejiang University, Hangzhou 310009, China; 7 Key Laboratory of Bioorganic Phosphorus Chemistry & Chemical Biology, Department of Chemistry, Tsinghua University, Beijing 100084, China; 8 Stoddart Institute of Molecular Science, Department of Chemistry, Zhejiang University, Hangzhou 310027, China; 9 Green Catalysis Center and College of Chemistry, Zhengzhou University, Zhengzhou 450001, China; 10 MOE Key Laboratory of Macromolecular Synthesis and Functionalization, Department of Polymer Science and Engineering, Zhejiang University, Hangzhou 310027, China

**Keywords:** CDs, supramolecular chemistry, theranostics, nanoparticles, nanomedicines

## Abstract

With the development of personalized medical demands for precise diagnosis, rational management and effective cancer treatment, supramolecular theranostic systems have received widespread attention due to their reversibly switchable structures, sensitive response to biological stimuli and integration ability for multiple capabilities in a single platform with a programmable fashion. Cyclodextrins (CDs), benefiting from their excellent characteristics, such as non-toxicity, easy modification, unique host–guest properties, good biocompatibility, etc., as building blocks, serve as an all-purpose strategy for the fabrication of a supramolecular cancer theranostics nanodevice that is capable of biosafety, controllability, functionality and programmability. This review focuses on the supramolecular systems of CD-bioimaging probes, CD-drugs, CD-genes, CD-proteins, CD-photosensitizers and CD-photothermal agents as well as multicomponent cooperation systems with regards to building a nanodevice with functions of diagnosis and (or) therapeutics of cancer treatment. By introducing several state-of-the-art examples, emphasis will be placed on the design of various functional modules, the supramolecular interaction strategies under the fantastic topological structures and the hidden “bridge” between their structures and therapeutic efficacy, aiming for further comprehension of the important role of a cyclodextrin-based nanoplatform in advancing supramolecular cancer theranostics.

## 1. Introduction

Cancer, accounting for nearly 19.3 million new cases and 10 million deaths in 2020 according to reports by the WHO [1], is a critical global public health problem and a leading cause of death with risks of significant damage to human life and health. Currently, traditional treatment methods, including surgery, chemotherapy and radiotherapy, cannot always meet the current clinical requirements for cancer treatment because of their limitations and serious side effects [2,3,4,5,6]. Cancer treatment still brings great challenges.

Supramolecular chemistry was recognized through the Nobel Prizes in Chemistry in 1987 and 2016 for “their development and use of molecules with highly selective interactions-specific structures” [7] and “the design and construction of molecular machines”, respectively. Supramolecular interactions, including Van de Waals, hydrophobic effects, host–guest interactions, metal-coordination and hydrogen bonding, contribute to holding supramolecular species together. Unlike conventional chemistry, the bond in supramolecular chemistry is non-covalent, suggesting that it is the result of thermodynamic equilibrium during assembly. It permeates many biological phenomena, from biological components, such as proteins with a three-dimensional structure, DNA with a double helix structure and cells with a lipid bilayer structure, to various physiological response processes (e.g., protein–DNA recognition by hydrogen bonding), enzymatic catalysis processes (e.g., hematin–iron as a coordinate complex) and drug-active receptors (e.g., substrate/response). These weak and reversible interactions are key to comprehending biological self-assembly systems, as well as the design and synthesis of supramolecular nanodevices with the following potential for cancer theranostics. First, they endow stimuli-responsive properties on nanodevices, which makes it possible to deliver therapeutic agents in space-, time- and dosage-controlled modes [8,9,10]. Second, to enhance the stability and circulation time of nanodevices in a physiological environment, strategies to strengthen weak forces are adopted: (a) preorganization of binding sites; (b) the hydrophobic cavity effect; (c) the multivalent effect and (d) synergy of multiple forces [11,12]. Third, owing to the modular feature of supramolecular chemistry, targeting components, diagnostic contrast agents, imaging probes and therapeutic agents can be ingeniously integrated into supramolecular building blocks by relying on non-covalent interactions. In a word, these non-covalent interaction properties play a critical role in nanodevices for cancer theranostics and confer reversible and stimuli-responsive properties on nanodevices with novel multimolecular supramolecular architectures of complexity and functionality.

The most widely used supramolecular hosts—macrocyclic molecules, including cyclodextrins (CDs), calix[n]arenes, cucurbit[n]urils and pillar[n]arenes, possess signature cavities, which could be employed as the building blocks of nanodevices through supramolecular technology in the realm of cancer theranostics [10,13]. On account of dynamic host–guest recognitions, rapid and safe therapeutic strategies with excellent outcomes are explored to confront limitations in effective cures by multiple causes such as poor solubility and stability, off-target property through leakage of guests to other cells, short circulation time, unknown toxicity and co-morbidities. Endogenous stimuli (pH, redox and enzymes, etc.) taking advantage of the differences between cancerous and normal cells/tissues in the microenvironment or non-invasive and remotely controlled exogenous stimuli (light, temperature, magnetic, voltage and molecular competition, etc.) are being exploited and developed to regulate supramolecular disassembly behavior for accurate diagnosis and therapy [14,15,16,17].

This paper shows a summary of the current advances in CD-based supramolecular architecture for intelligent bioimaging and molecular recognition, controlled drug delivery, protein and gene delivery, photodynamic/photothermal therapy and other applications, including ingenious structures, unique strategies and stimulation–response controlled release (light response, pH response, REDOX response and multiple responses). A deep understanding of progression in supramolecular CD-based cancer therapy and diagnosis offers further insights into new therapeutic strategies.

## 2. Cyclodextrins

**Figure 1:**
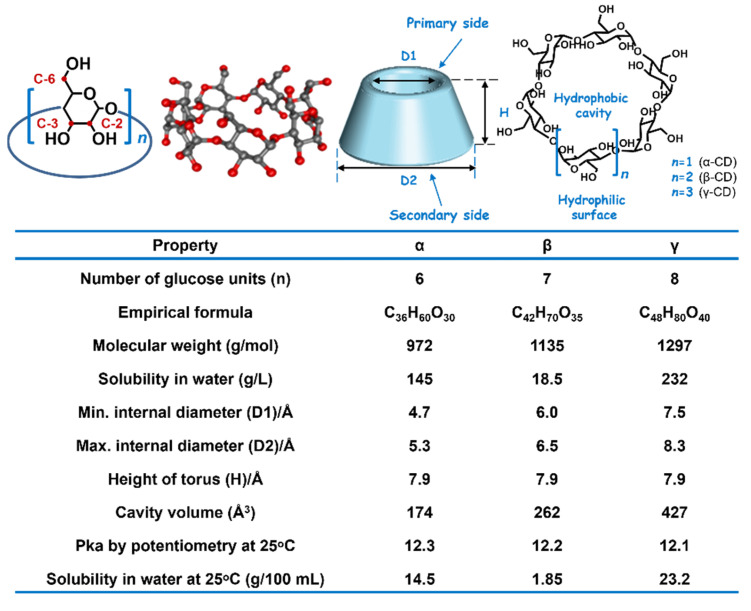
Chemical structures and parameters of CDs [28,29].

Cyclodextrin (CD), which was accidentally discovered by Anthony Villiers over 100 years ago as a byproduct of enzymolysis of potato starch by bacillus, is a class of cyclic oligomers with 6, 7, 8 glucopyranose units via-1, 4-D-glycosidic bonds corresponding to α-, β- and γ-CD, respectively (Figure 1). CDs consist of two hydrophilic shells and a hydrophobic cavity. The external part with abundant hydroxyl groups is not only beneficial to improving the solubility of CD but can also be modified selectively into diverse functional moieties to confer CD a tailored functionality and satisfy different physiochemical environments [18]. The interior cavity, chiral and less polar [19], can readily form an inclusion complex through a relatively isolated microenvironment to encapsulate various guest molecules, such as azobenzene, adamantane, ferrocene, cholesterol, photosensitizer, antidrug, etc. In a nutshell, CD is a promising candidate to enhance water solubility and stability of hydrophobic guest molecules. Because of versatile sorts of CD derivatives, many CD-based supramolecular systems with manifold approaches have been exploited. The fields covered include food and pharmacy [20], catalysis [21], analytical chemistry (such as separation [22], sensing [23,24], materials science [25,26,27]) and others.

## 3. Cyclodextrins’ Advantage in Cancer Theranostics

**Figure 2:**
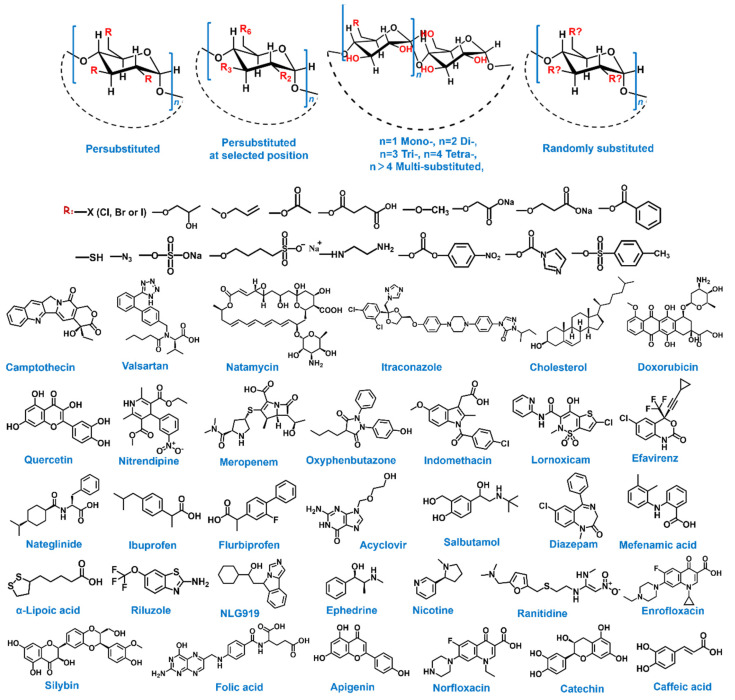
Schematic representation of the multi-functionalized derivative types of CD (**up**) and the typical and new guests for CDs family (**down**).

What is more, scientists investigate CDs for their performance in theranostics and biological applications due to the following unique properties: (1) Natural: CDs are produced as a result of the enzymolysis of starches. This natural availability makes CDs highly biodegradable, greatly biocompatible and non-toxic toward biological systems, far beyond that of other supramolecular hosts. Multiple CD-including pharmaceutical compounds have successfully been approved by the European Medicines Agency (EMA) and the Food and Drug Administration (FDA) [30,31,32]; (2) Versatile functionalization: the hydroxy groups of CD are modified not only to different positions but also into various functional groups (such as alkyl chains [33], sulfhydryl moieties [34], poly(ethylene imine) [35], fluorophore [36,37], photothermal agent [38], PEG chains [39], polymers [40] and co-polymers [41]) to meet diverse requirements and specific environments of cancer therapy (Figure 2, up); (3) Superior host ability: (a) despite having the same cavity depth, the internal diameter enlarges with the number of D-glucose units, suggesting that manifold guest molecules can be encapsulated in three hosts depending on their spatial size. New guest molecules exploited in theranostics and biological applications comprise anticancer drugs (camptothecin [42]), pathway inhibitors (NLG919 [43]), amino acids [44], steroids [45], natural products (curcumin [46]), etc. [47,48,49] (Figure 2, down). (b) The hydrophobic cavity of CD provides a space to enhance the solubility of the loaded reagents, as well as prevents the enzymatic hydrolysis process to maintain its biological activity [50]. (c) The therapeutic agent could be non-covalently conjugated through the host–guest inclusion complex, which stabilizes in the physiological environment and is emitted upon reaching a specific location.

## 4. Biomedical Applications

### 4.1. Bioimaging and Molecular Recognition

**Figure 3:**
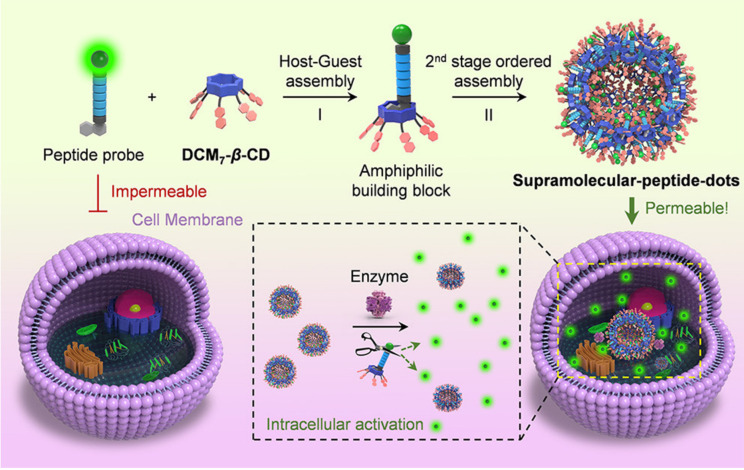
Schematic illustration of CD-based supramolecular peptide self-assemblies (Spds) with improved cellular delivery and spatiotemporal imaging. Reproduced with permission from [56], copyright 2020 American Chemical Society.

Visualization of biological events in living animals provides necessary information for cancer diagnosis, which has become one of the hot fields in biology and chemistry. The characteristics of supramolecular interaction enable CDs to incorporate targeting components and imaging moieties, presenting good imaging characteristics and multimodule imaging capability. Moreover, imaging time and excretion behavior can be optimized with tunable cavity sizes of CD [51,52,53,54]. Liu et al. designed a hypoxia fluorescence probe composed of sulfato-β-CD, azobenzene derivative and green-fluorescent dye, which self-assembled by electronic interaction and hydrophobic interaction [55]. The system turns “off” by the supramolecular assembly skeleton to quench the fluorescence with the photoinduced electron transfer (PET) mechanism; the system turns “on” by fracture of the nitrogen-nitrogen double bond of the hypoxic-responsive azobenzene group through a reduction reaction to restore the fluorescence; hypoxic cell imaging was achieved by these fluorescence changes. The ternary system provided a new path for the non-covalent formation of hypoxic-trigger fluorescent probes. Biomarker-activated bioimaging systems show great potential in therapeutic and diagnosis due to their high biological substrate specificity. Tian, He and co-workers constructed CD-based peptide self-assemblies with hierarchical supramolecular architectures which strengthen peptide-associated fluorescence imaging and antimicrobial efficacy [56] (Figure 3). In this system, the fluorescent peptides or the antimicrobial peptide successfully achieved intracellular delivery through non-covalent conjugation to hepta-dicyanomethylene-4H-pyran appended β-CDs via a host–guest inclusion complex, which monitored the apoptosis mediators and process of mitosis using spatiotemporal imaging or enhanced the antimicrobial efficacy. The Spds platform integrated a mixture of intelligent potentials, such as peptide delivery, targeting, visualizations and antimicrobial efficacy of known therapeutic peptides. This study offered new insight to solve the limitations of functional peptide applications.

Near-infrared (NIR) has rapidly become a highly attractive optical region in biological imaging technologies because of its merits of low signal interference, great penetration depth and spatial resolution. Pu and co-workers designed a renal-clearable CD-based supramolecular reporter (CyP1) for NIR fluorescence imaging-guided bladder cancer therapy [57]. CyP1 includes an alkyne-functionalized HPβCD, a NIR signaling moiety and an aminopeptidase N (APN)-activated substrate. HPβCD could act as a supramolecular pocket to encapsulate a biomarker-reactive moiety in its cavity through hydrophobic interaction and could function as a renal-clearable enabler to induce CyP1 to accumulate efficiently in the bladder, specifically reporting the APN level to synchronize bladder cancer-related information. This study may have certain significance for fabrication of renal-clearable reporters for real-time tracking of other diseases. An example of applications to other diseases was published by the same research group [37]. Based on the same core design principle, this group expanded the application of a supramolecular probe based on CD by adjusting biomarkers according to different detection objects. Bioimaging in the NIR-II window for the precise monitoring of SARS-CoV-2 was established. The macromolecular prober of β-CD modified hemicyanine fluorophore-a protease peptide substrate (SARS-CyCD) was exploited. The imaging element self-caged by linking the peptide substrate, resulting in a “turn-off” state; and was caged out by the enzymatic cleavage linking bond, resulting in a “turn-on” state. Excellent detection outcomes were shown in the lungs of SARS-CoV-2 Mpro-positive mice. Moreover, on account of the high renal-clearance efficiency, SARS-CoV-2 infection was assessed by analysis of activated CyCD in urine.

### 4.2. Nanodevices for Controlled Drug Delivery

Drug delivery systems (DDS) relate to a method that could preserve and transport the demanded dose of the therapeutic reagent to the required receptor sites or targets for drug release and absorption. The aim is usually to achieve maximum therapeutic efficacy of therapeutic agents and to overcome their limitations such as poor stability and solubility, lack of selectivity, bad bioavailability, drug aggregation, bad biodistribution and adverse reactions due to inappropriate disposition [9,58,59]. A therapeutic approach that combines drug delivery with supramolecular systems has shown great promise in recent years, which benefits from the unique physicochemical attributes of supramolecular nano-assemblies, including size, high surface-to-volume ratio and shapes [18,60,61]. Through the design and synthesis of CD derivatives, as well as CD-based supramolecular self-assembly, nanomedicine can be fabricated using a variety of architectures with unique topological characteristics, including polymeric nanoparticles (NPs), (pesudo)rotaxanes, catenanes, dendrimers, cross-linked networks, micelles, liposomes, etc. [62,63,64,65,66,67,68]. In addition, nanomedicine can be prepared by hybridization of CD with various inorganic ingredients, for example, mesoporous silica nanoparticles (MSNs) [69], metal-organic frameworks (MOFs) [70,71] and graphene oxide (GO) [72]. Below, some examples of supramolecular nanomedicine that have demonstrated promising prospects in cancer therapy and diagnosis are discussed.

By taking full advantage of CD-based supramolecular chemistry, anticancer drugs such as doxorubicin (DOX), cisplatin (Pt), paclitaxel (PTX), tamoxifen (TAM), temozolomide (TMZ) and camptothecin (CPT) have achieved great controlled release through the formation of host–guest complexes in the physiological environment [46,73,74,75]. Great studies based on the supramolecular system are too numerous to mention. Xu and co-workers [76] prepared supramolecular nanocages by covalently cross-linking multiple β-CD molecules via GSH-responsive bonds. DOX was stably encapsulated via host–guest interaction and hydrogen bonds between β-CD units, and further was specifically released by tons of disulfide bond cleavage in the tumor microenvironment. Additionally, GSH depletion induces the disturbance of the redox equilibrium in tumor cells, which sensitizes DOX-based chemotherapy effects and triggers immunogenic cell death (ICD) to realize stronger tumor inhibition and prolonged survival. The CD nanocage enriches the range of delivery vehicles for cancer therapy.

**Figure 4:**
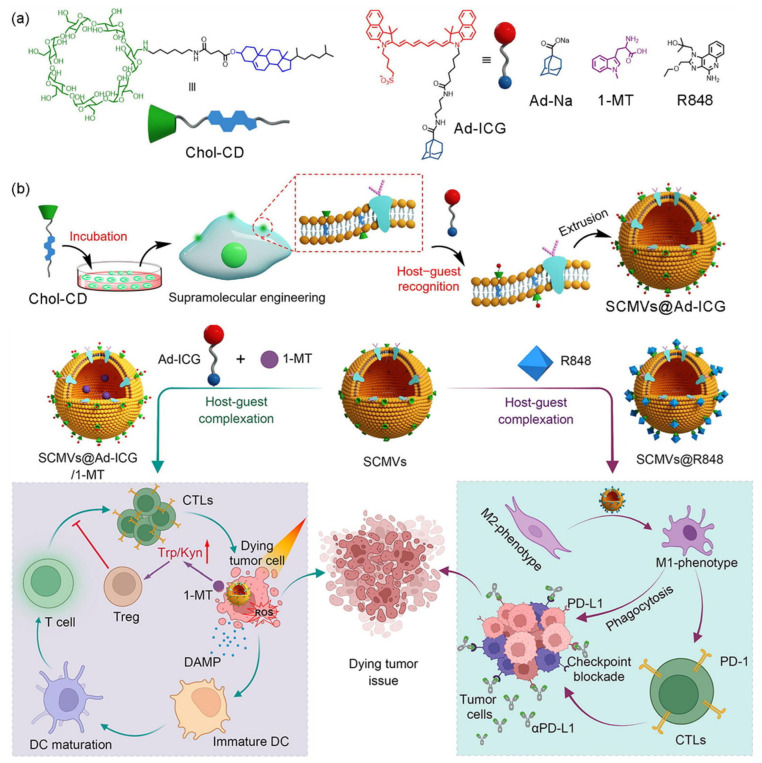
Fabrication of SCMVs (chemical structures (**a**) and illustration of the general preparation (**b**)). Schematic illustration of CD-based supramolecular cell membrane vesicles (SCMVs) with co-delivery of indocyanine green and 1-methyl tryptophan (1-MT) for cancer photodynamic therapy and immunotherapy (**left**); and resiquimod (R848) for immune checkpoint blockade therapy (**right**). Reproduced with permission from [77], copyright 2022 Elsevier.

Another new delivery vesicle was published by Qi et al. [77] (Figure 4). They prepared cell-friendly supramolecular membrane vesicles (SCMVs) by specific binding of the cell membrane to CD-based cholesteryl where cholesterol self-inserted into a phospholipid bilayer with its polar hydroxyl group. The SCMVs successfully encapsulated a clinically approved dye through the host–guest interaction from β-CD-adamantane, which is capable of near-infrared imaging and photodynamic therapy (PDT). Intriguingly, an indoleamine 2,3-dioxygenase (IDO) inhibitor was loaded by a SCMVs-mediated host–guest inclusion complex, allowing PDT and immunotherapy. Toll-like receptor 7 and 8 (TLR7/8) agonist was also delivered, showing a high M2/M1 tumor-associated macrophage ratio. This supramolecular technology approach provides a universal and cell-friendly strategy for the development of living cell-involved nanomaterials for precise cancer therapy.

To cope with the complex tumor environment, scientists have adopted multiple therapeutic modalities such as a combination of chemotherapy and chemodynamic therapy. Wang and co-workers [78] constructed H_2_O_2_-responsive nanoparticles by a one-pot supramolecular polymerization-induced self-assembly, which included platinum (IV)-prodrug-conjugated ferrocene-modified CD and PEG-ferrocene terminator and were driven by the host–guest interactions of the β-CD-Fc inclusion complex and the size regulation effect of PEG segments. When the nanoparticles were exposed to the tumor microenvironment, on the one hand, ferrocene acted as a catalyst to produce cytotoxic hydroxyl radicals from intracellular hydrogen peroxide through a Fenton-like reaction, and on the other hand, the prodrugs were reduced to cisplatin, thus jointly promoting disassociation of supramolecular structure. This self-augmented cascade chemodynamic therapy and chemotherapy enhanced the therapeutic outcomes. Yang et al. adopted a similar strategy [79]. They developed a hybrid supramolecular polymeric nanomedicine (SNPs), in which the CPT guest and β-CD host linked via ROS-responsive thioketal bonding (CD-S-CPT), and iron ions coordinated with carboxylate acid groups on the functionalized β-CD. After SNPs were internalized by the cancer cells, overexpressed hydrogen peroxide converted into highly toxic hydroxyl radicals through a Fenton reaction, which further cleaved the thioketal moiety to release the antidrug, achieving cascade-amplified therapeutic efficacy by combining chemotherapy and chemodynamic therapy. Moreover, this combination therapy triggered a strong ICD effect to rebuild the immuno-microenvironment of the tumor. More importantly, the introduction of an immune checkpoint blockade (PD-L1) augmented immune therapy and long-term inhibition of tumors.

Image-mediated tumor synergistic therapy—“theranostic”—shows great potential in personalized medicine, real-time monitoring of the nanomedicine therapy process and feedback of the nanomedicine therapy effect. Chen and co-workers [42] presented a theranostic supramolecular polymer self-assembled by the host–guest interaction of monomers, in which a camptothecin (CPT) guest was conjugated with a β-CD host via GSH-triggered bonding. The assembly of the supramolecular polymer not only resulted in a significant 232-fold increase in the solubility of CPT but also perfectly maintained the antitumor activity of its lactone form under physiological conditions. Considering the stability and the property of targeting (RGD moiety) and imaging (^64^Cu-chelated NOTA moiety), the introduction of a polymer terminated by CPT was self-assembled in an orthogonal manner to form a nano-platform which was driven by a hydrogen bond, π-π interactions and host–guest interactions. This nanomedicine demonstrated high anticancer properties and unnoticeable long-term immunotoxicity. The same authors also developed a “supramolecular gate”-controlled nanotheranostic platform inspired by the combination of supramolecular chemistry and mechanically interlocking molecules [80]. The platform with a core-shell crosslinked structure was constructed by using a β-CD-based polyrotaxane skeleton and two stoppers (a photothermal agent perylene diimide (PDI) and a targeting ligand RGD), which was exerted to encapsulate paclitaxel. The supramolecular nanoparticles with suitable sizes, high stability and high drug encapsulation capacity were a result of the hydrophobic interactions between poly(ε-caprolactone) segments and CDs and the π-π interactions from the PDI. Interestingly, the drugs locked without leakage during the circulation process benefited from the ingenious supramolecular structure; the drug specifically released profited from the high-concentration GSH cleavage disulfide bond. The integrated system of delivery, targeting, imaging and photothermal therapy shows excellent therapeutic efficacy against orthotopic breast cancer and lung metastasis.

### 4.3. Nanodevices for Gene and Protein Delivery

**Figure 5:**
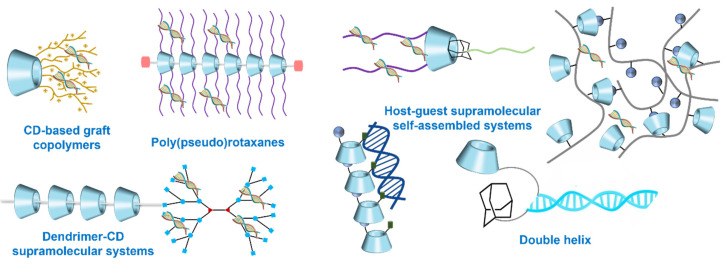
Schematic representation of CD-based gene delivery systems.

In addition to their use in chemotherapeutic delivery, CD-based supramolecular assemblies are directed to transport genetic therapeutic agents (Figure 5). Significantly, these supramolecular structures present low material toxicity, high transfection efficiency, great stability in the process of transport and controllable gene release rates by regulating CD concentration [49,81,82].

Typically, Sollogoub’s group constructed an adamantane-modified bridged CD derivative (CD1) that prevented self-inclusion and head-to-head and could enable supramolecular polymerization and cooperative assembly with DNA. In this work, the bridge with the adamantyl group was attached to the β-CD unit via ammonium groups, which improved solubility and promoted interaction with DNA. The monomer CD1 could build supramolecular polymers at millimolar concentrations. This self-assembling behavior was also demonstrated at micromolar concentrations in the presence of DNA, resulting in a significant ability to compact DNA via cooperative interactions. Finally, the author successfully expanded DNA compaction ability to siRNA transfection in gene silencing [83].

Combination therapy, in which anti-tumor drugs and genes are delivered simultaneously, provides a simple and effective manner to the treatment of cancer. Li, Wu and co-workers designed amphiphilic supramolecular polymeric micelles in which two β-CD-based polymeric complexes as hydrophobic cavities enclose chemotherapeutic DOX and the Nur77ΔDBD gene [84]. The high transfection effect of the gene and fast cellular uptake of DOX overcome dual drug resistance mechanisms to achieve significantly synergistic inhibition of tumor cell viability. Zarghami’s group [85] also utilized a similar tactic to achieve efficient and targeted co-delivery of DOX and siRNA. In recent years, many reports have shown that CDs acting as supramolecular building blocks could be utilized to be gene delivery systems and co-delivery systems (Table 1).

**Figure 6:**
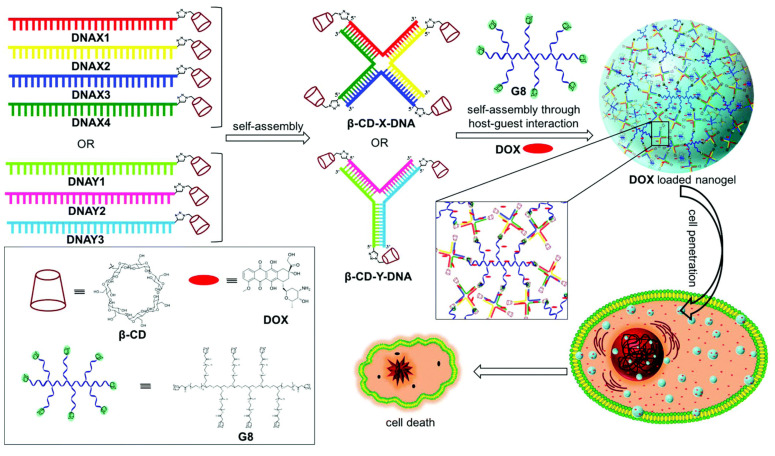
Schematic illustration of CD-based-size controllable DNA nanogels from the self-assembly of β-CD functionalized branched DNA nanostructures with a star-shaped adamantyl-terminated 8-arm poly(ethylene glycol) (PEG) polymer via a host–guest complex for antitumor drug delivery. Reproduced with permission from [99], copyright 2018 Royal Society of Chemistry.

In addition to serving as therapeutic agents, genes may also be potential candidates to construct nanodevices for drug-delivery applications. Varghese et al. developed DNA nanogels using supramolecular assembly of β-CD functionalized branched DNA nanostructures with a star-shaped adamantyl-terminated 8-arm poly(ethylene glycol) (PEG) polymer via multivalent host–guest interactions. Nanogels with excellent cell permeability could be obtained by controlling the concentration of the guest and host at a low level and exhibited great biocompatibility and highly efficient drug-loading ability, which indicated that nanogels are promising drug delivery vehicles. This DNA-mediated drug delivery system provided new possibilities for cancer therapy [99] (Figure 6).

Protein drugs are emerging. Meanwhile, the intrinsic characteristic of proteins, such as large size, short circulation time, fragile tertiary structure, enzyme degradation and low permeability parameters, pose huge obstacles to their clinical application. To solve these problems, the development of a novel oral protein delivery system based on chitosan (CS) nanoparticles and the ovalbumin (OVA)-CD inclusion complex was presented by Gao et al. [100]. Due to non-covalent interactions, the OVA-loaded CD/CS nanoparticles exhibited suitable particle size, high efficiency of protein encapsulation and a long-acting release rate in vitro. The nanoparticles not only act as a promising delivery vesicle for oral antigen but also as an inducer of the immune response of the intestinal mucosa. Another delivery system with excellent performance for different proteins was reported by Feng et al. [101]. They developed a multiarmed amphiphilic CDs (CDEH)-based novel self-assembled protein delivery platform that was driven by a multivalent electrostatic interaction and a hydrophobic interaction. Self-assembled nanoparticles with excellent protein-loading capacity can be prepared only by simple mixing of CDEH with proteins, achieving the maximum preservation of protein activity. Furthermore, the targeting aptamers could be integrated into nanoparticles by the introduction of targeting-group-modified adamantane via a host–guest interaction, leading to superior accumulation in tumors. AS1411-aptamer-targeted CDEH nanoparticles efficiently inhibit tumor progression as a result of intracellular saporin-specific delivery; folate-targeting CDEH nanoparticles effectively suppress tumor growth on account of joint Cas9 protein and Plk1-targeting sgRNA-selected delivery. The study offered a novel approach to protein delivery.

### 4.4. Photodynamic Therapy (PDT)

Photodynamic therapy (PDT) is a treatment method based on photochemistry using light combined with photosensitizers. The principle is to activate photosensitizers to convert light energy into highly cytotoxic reactive oxygen species or free radicals which can induce cell death. Non-invasiveness, high cure rate and few side effects are the most attractive features of PDT, which became one of the alternatives to traditional cancer treatment. Traditional organic photosensitizers are “fat-loving” molecules with large π-conjugated structures such as porphyrin, chlorin e6 (Ce6) and phthalocyanine derivatives. They are limited by bad water solubility, low selectivity, photocytotoxicity and lack of tumor targets [102]. Great effort has been made to overcome these drawbacks (Table 2). Liu Yu’s group constructed a supramolecular system of multivalent assemblies of sulfonated aluminum phthalocyanine (PcS), an adamantane-connected pyrenyl pyridinium derivative (APA2) and folic acid-modified β-CD (FA–CD). The nanoparticle was prepared in two steps: in the first step, PcS could associate with APA2 to form non-fluorescent irregular binary nanoaggregates that integrate π–π interactions, electrostatic interactions and the heterodimer aggregation quenching effect; in the second step, FA-CD could co-assemble with a binary PcS/APA2 supramolecular complex via the host–guest interaction between CD and adamantane to obtain a ternary system with great biocompatibility and targeting capacity of cancer cells. The nanoparticle could allow for the specific accumulation in Hela cells by folic-acid-mediated endocytosis and promote efficient generation of singlet oxygen, thereby exhibiting a high photodynamic effect. Intriguingly, the system adaptively depolymerized in an intracellular physiological environment of Hela cells and the PcS component encountered the autonomous translocation process from the lysosome to the nucleus, which could facilitate timely monitoring of the apoptosis process. This strategy would provide new opportunities for precise organelle-oriented theranostic systems [103].

### 4.5. Photothermal Therapy (PTT)

Photothermal therapy (PTT), another type of phototherapy, is also widely used in the treatment of cancer. Since CDs themselves have no photothermal properties, owing to their supramolecular interaction specificity, they serve as a connecting component of sophisticated surface modification to assist molecules to anchor on the nanoplatforms surface of the PTT therapy system involving CDs. The collaboration materials involve gold nanomaterials (nanorods, nanoshells [109], nanoflower [110], etc.), graphene oxide (GO) [72], magnetic nanoparticle hybrid nanomaterials [111], zeolitic imidazolate framework-8 (ZIF-8) [112], platinum nanoparticles [113], etc. In recent years, a large amount of CDs-based supramolecular self-assembly/hybrid systems for PTT have been studied (Table 3).

Ping et al. [118] prepared a multifunctional gold nanorod (AR@PSS@PCM, termed as ANP) with a layer-by-layer self-assembly strategy, where the zero layer was cetyltrimethylammonium bromide (CTAB)-mediated-Au nanorods (ARs); the first layer introduced polystyrene sulfonate to enhance biocompatibility (PSS); the second layer introduced a supramolecular polymer (PCM), which was self-assembled by the host–guest interaction of polyethylenimide modified β-CD and adamantane derivatives. The ANP could not only act as a container to transport CRISPR or Cas9 protein capable of targeted disruption of PD-L1 but also serve as a photothermal converter in the NIR-II window to trigger ICD and gene expression of Cas9. This therapeutic strategy for synergistic immune checkpoint blockade and ICD activation could reverse the immunosuppressive environment, thus presenting high inhibition effects against primary and metastatic tumors and long-term immune memory effects against recurrent tumors (adjustment sequence).

**Figure 7:**
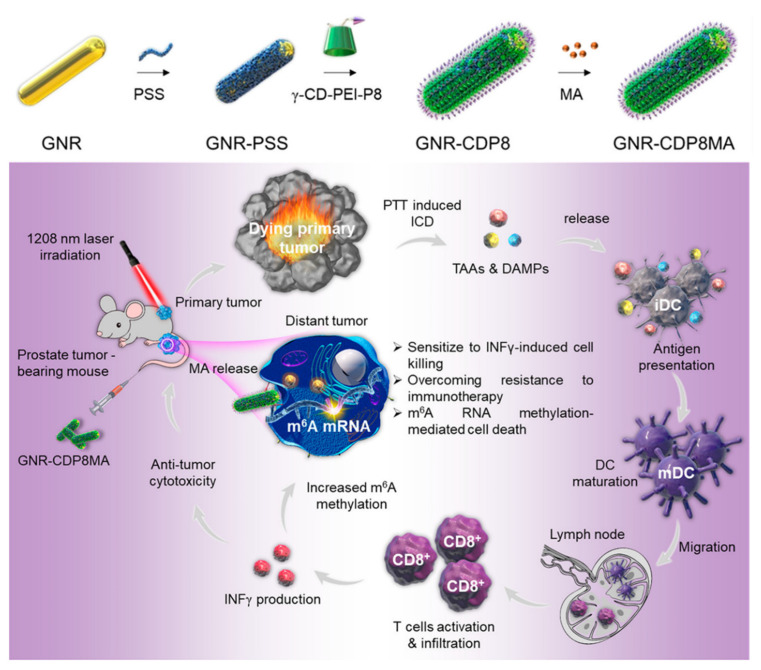
Schematic illustration of CD-functionalized gold nanorods (GNR-CDP8) with meclofenamic acid (MA) for NIR photothermal immunotherapy in prostate cancer. Reproduced with permission from [119], copyright 2022 American Chemical Society.

Another example of a CD-mediated supramolecular cationic gold nanorod was reported by Yan et al. (Figure 7) [119], presenting a nanoplatform based on CD-functionalized gold nanorods (GNR-CDP8MA). Benefiting from electrostatic interactions, GNRs were coated with a layer of negatively charged polystyrenesulfonate polymers (PSS) and then were assembled with a γ-CD-based cationic polymer (γ-CD-PEI-P8) conjugated with ab 8-mer peptide (GRPR) targeting a prostate tumor; benefiting from hydrophobic interactions, a m^6^A RNA demethylase inhibitor was encapsulated in the cavity of CD. This nanoplatform could be exclusively taken up by prostate tumor cells and achieved a high drug release rate due to its photothermal conversion ability, as well as PTT-mediated ICD. A combination of m^6^A RNA methylation with second near-infrared photothermal therapy could synergistically achieve immunotherapeutic eradication of tumor cells.

## 5. Deficiencies of Current CD-Mediated Cancer Theranostics

In spite of the fact that much significant progress has been made related to the supramolecular systems of CD with great therapeutic efficacy and biocompatibility, some limitations and challenges still exist. (1) Although CD is a natural product, hybrid/supramolecular nanodevices based on CD are artificial compounds, and immunotoxicity is unavoidable. (2) The stability of supramolecular systems needs more precise criteria to avoid premature release, incomplete release or inability to release the loaded cargoes, achieving a balance between instability and stability resulting from the unique non-covalent bond interactions of supramolecular chemistry. (3) Recently, the functionalization of CD usually adopts random/mono-substitution on the primary rim or secondary rim. While some groups [120,121] have explored region-selective modification of CDs, the complexity of synthesis, the low yield and the difficulty of scaling up the production of CDs require further efforts for biological applications. (4) Few supramolecular systems have been reported to exhibit subcellular (nucleus, lysosome, mitochondria and others) targeting ability, and they still mainly target the cell membrane surface. (5) The variety of biological stimuli for supramolecular systems needs to be further explored and expanded.

## 6. Conclusions

In conclusion, the recent progress in the bio-applications of CD-based supramolecular systems has been highlighted. Convenient features of CD, including natural products, easy functionalization, well-known binding constants to diverse guests and excellent biocompatibility, have been extensively developed and received widespread attention for the construction of novel multifunctional supramolecular biomaterials. Benefiting from supramolecular chemistry, CDs, pharmaceutical excipients, efficiently enhance the stability/solubility of anti-tumor drugs or other agents (proteins, DNA, photosensitizers, etc.), to improve the pharmacokinetic performance and boost the duration of activity. On the basis of its dynamic nature, the resultant functional supramolecular biomaterials exhibit feasible functionalization through “Lego-like” self-assembly, reversibly convertible structures and intelligent responsiveness to a variety of biological stimuli, thus fulfilling the requirements during the cancer therapy and diagnosis process. In fact, the use of CDs as a unique technological strategy has been unable to meet the needs of cancer treatment, as it has been recognized that there are many advantages to integrating several different technological approaches/materials with CD-based systems into supramolecular theranostic systems. The class of CD-based nanodevices includes the CD-based supramolecular self-assembly system and the hybridization of CD-based supramolecular components with solid materials.

Given the complexity of tumor therapy, experts from different research fields such as tumor biology, pharmacology, chemistry, materials science and nano-engineering should collaborate comprehensively and vigorously to create and fabricate a combination therapy system. In conclusion, this paper emphasizes that supramolecular self-assembly systems based on CD open a new way of thinking in the development of intelligent and effective nanotherapeutic systems.

## Figures and Tables

**Table 1:** Basic information of CD-based gene delivery systems and co-delivery systems.

Guest	Other Components	Supramolecular System	Therapeutic Agents	Ref.
adamantane		double helix	DNA	[86]
PEI	2,4-dinitrobenzaldehyde	polyrotaxanes	DNA	[87]
adamantane	PAMAM dendrimer	bilayer supramolecular sphere	DNA	[88]
PLLD-Arg	PEG	supramolecular hydrogel	pMMP-9	[89]
ferrocene	PEI, PEG	supramolecular aggregate	pMMP-9	[90]
adamantane	folate acid, PEI, PEG	supramolecular self-assembly	pDNA	[91]
azobenzene	dextran	supramolecular self-assembly	DNA	[92]
adamantane	pDMAEMA, pMPC	phospholipid bilayer	DNA	[93]
adamantane	PEI, DOX	supramolecular self-assemble	p53, DOX	[94]
DTX	folate acid, DSPE, PEG	supramolecular self-assembly	DTX, siRNA	[95]
adamantane	MSN, PGEA, IPTS	supramolecular hybrid system	pDNA, siDNA, DOX	[96]
monensin	PEI, PGA	supramolecular self-assembly	DNA, TRAIL	[97]
DOX	PDMAEMA, PEG	supramolecular self-assembly	DOX, miR-122	[98]

**Table 2:** Basic information of CD-based PDT.

Guest	Other Components	Supramolecular System	PDT Type	Combined Therapy	Ref.
ferrocene	HA, poly (γ-glutamic acid), TGF-β1 inhibitor	LC@HCDFC NPs	Ce6	immunotherapy	[104]
PEG	NO donor	α-CD-Ce6-NO NPs	Ce6	chemotherapy	[105]
adamantane	AuNR, MSN, PEG, peptide	AuNR@MSN-CD/Ada-RLA/CS(DMA)-PEG	ICG	PTT	[106]
Ce6	CS, PEG	CS–CD–Ce6	Ce6	immunotherapy	[107]
CPT	UCNPs, lactobionic acid, PEG, ROS-generating agent	UCNP@mSiO_2_-NBCCPT@(FITC)/β-CD-PEG-LA@DHMA	UCNPs	chemotherapy	[108]

**Table 3:** Basic information of CD-based PTT.

Guest	Other Components	Supramolecular System	PTT Type	Combined Therapy	Ref.
pyridine-2-imine	AuNPs, DNA	Au-DNA-αCDs	AuNPs	photoacoustic imaging	[114]
azobenzene	transferrin, polylysine, PEG	TPM-Azo⊂GOCD	GO	mitochondrial physical disruption	[72]
paclitaxel	HA, TPP	FNPs-pDA@HA-TPP-CD-PTX	Polydopamine	chemotherapy	[115]
adamantane	RGD, DOX	CuS-DOX	CuS	chemotherapy	[116]
adamantane	Dextran, Fe_3_O_4_, PGEA, p53	Fe_3_O_4_@Dex-PGEA	Fe_3_O_4_	magnetolytic therapy	[117]

## Data Availability

The data presented in this study are all from published papers and can be obtained from references.
